# Shedding light on biochemical changes in single neuron-like pheochromocytoma cells following exposure to synchrotron sourced terahertz radiation using synchrotron source Fourier transform infrared microspectroscopy

**DOI:** 10.1107/S1600577524010944

**Published:** 2025-01-01

**Authors:** Palalle G. Tharushi Perera, Jitraporn Vongsvivut, Denver Linklater, Zoltan Vilagosh, Dominique Appadoo, The Hong Phong Nguyen, Mark J. Tobin, Rodney Croft, Elena P. Ivanova

**Affiliations:** a School of Science RMIT University 2476 Melbourne Victoria3001 Australia; b School of Science, Computing and Engineering Swinburne University of Technology Melbourne Victoria3122 Australia; c IR Microspectroscopy (IRM) Beamline ANSTO-Australian Synchrotron 800 Blackburn Road Clayton Victoria3168 Australia; d Biomedical engineering, Faculty of engineering and Information technology University of Melbourne Melbourne Victoria3010 Australia; e THz Beamline Australian Synchrotron 800 Blackburn Road Melbourne Victoria3168 Australia; f School of Psychology, Illawara and Medical Research Institute University of Wollongong Wollongong New South Wales2522 Australia; University College London, United Kingdom

**Keywords:** synchrotron source FTIR, principal component analysis, PCA, synchrotron source THz radiation, PC 12 neuronal cells, membrane permeability, blebs, synchrotron source Fourier transform infrared microspectroscopy, pheochromocytoma cells

## Abstract

Synchrotron sourced Fourier transform infrared (SS FTIR) microspectroscopy was employed to investigate the biological effects on the neuron-like pheochromocytoma (PC 12) cells after exposure to synchrotron sourced terahertz (SS THz) radiation. Over 10 min of exposure, the PC 12 cells received a total energy of 600 J m^2^, with a total incident power density of ∼1.0 W m^−2^ (0.10 mW cm^−2^) at the beam extraction port (BEP) of the THz beamline at the Australian Synchrotron. To investigate the metabolic response of PC 12 cells after synchrotron THz radiation exposure, we utilized the FTIR microscope at the Infrared Microspectroscopy IRM beamline, which offers high photon flux and diffraction-limited spatial resolution enabling the detection of functional group variations in biological molecules at a single-cell level. Principal component analysis (PCA) based on the SS FTIR spectral data revealed a distinct separation of SS THz-exposed and control (non-exposed) cells. According to the PCA loadings, the key changes in the exposed cells involved lipid and protein compositions as indicated by the stretching vibrations of CH_2_/CH_3_ groups and amide I/II bands, respectively. An increase in lipids, such as cholesterol, or notable changes in their compositions and in some protein secondary structures were observed in the SS THz-exposed cells. The PCA analysis further suggests that PC 12 cells might maintain cell membrane stability after SS THz irradiation through higher volumes of cholesterol and cell morphology via regulation of the synthesis of cytoskeleton proteins such as actin-related proteins. The outcome of this study re-emphasized the exceptional SS FTIR capability to perform single-cell analysis directly, providing (i) unique biological information on cell variability within the population as well as between different groups, and (ii) evidence of molecular changes in the exposed cells that could lead to a deeper understanding of the effect of THz exposure at a single-cell level.

## Abstract 2

This work presents an analysis of biochemical changes in pheochromocytoma cells in response to synchrotron sourced terahertz radiation using synchrotron sourced Fourier transform infrared microspectroscopy.

## 1. Introduction

Fourier transform infrared (FTIR) microspectroscopy has emerged as a feasible alternative to traditional techniques and has found utility in the medical and biological fields, especially in the study of cells, lipid compositions and secondary structure of proteins, such as protein misfolding along with *in vitro* aggregation (Westerink & Ewing, 2008 ▸; Vaudry *et al.*, 2002 ▸; Greene, 1978 ▸). Over recent years, synchrotron sourced (SS) FTIR microspectroscopy has been employed by many research groups owing to its high brilliance with 100–1000 times higher brightness than that of a Globar IR source in most laboratory based FTIR instruments, allowing micrometre-sized materials such as single, individual cells to be probed (Greene, 1978 ▸; Zhang *et al.*, 2018 ▸). In this work, we used SS FTIR microspectroscopy to gain insights into changes that may be triggered in major classes of biological molecules in PC 12 cells in response to exposure to SS terahertz (THz) radiation.

PC 12 is a clonal cell line, selected for this study, derived from the adrenal medulla of rats (Westerink & Ewing, 2008 ▸). This cell line is used as a typical neural cell line model, as PC 12 cells are capable of undergoing striking morphological and physiological changes under various conditions (Vaudry *et al.*, 2002 ▸; Greene, 1978 ▸; Zhang *et al.*, 2018 ▸). PC 12 cells are referred to as having neuron-like properties due to their ability to respond to nerve growth factor (NGF) by differentiating into sympathetic ganglion neurons (Das *et al.*, 2004 ▸) and they can be sub-cultured indefinitely (Westerink & Ewing, 2008 ▸). On treatment with NGF, PC 12 cells cease proliferation, initiate the process of differentiation by extending neurites while becoming electrically excitable (Das *et al.*, 2004 ▸). Most studies that address the mechanism of PC 12 cell differentiation in response to NGF monitor its effect in an exponentially growing cell population, assuming that every cell present in the population responds in an identical manner (Rudkin *et al.*, 1989 ▸). PC 12 cells have been used as a valuable model in studying cell signalling because the cells are also able to grow without some growth factors, hormones and neurotrophins, while exhibiting distinct responses to differentiation, cellular proliferation and survival which can be assessed independently (Vaudry *et al.*, 2002 ▸). PC 12 cells have also been widely used to study the neuroprotective effects from drugs and for components present in traditional Chinese medicine with neuroprotective agents or models injured due to oxidative stress (Zhang *et al.*, 2018 ▸).

The THz spectral range of electromagnetic radiation falls between microwave and infrared frequencies. The THz region can be defined as having a frequency of 0.1 to 20 THz (wavelengths of 3.0 mm to 15 µm, photon energies of 0.413 meV to 82.6 meV) (Vilagosh *et al.*, 2022 ▸). THz radiation is non-ionizing, and has no capacity to break hydrogen bonds, yet has the required energy to interact with van der Waals forces (Sun *et al.*, 2017 ▸; Parsegian, 2005 ▸). With the development of ultrafast laser technology, THz technology has made unprecedented progress in application fields including security testing, communication and especially in biomedicine (Liu *et al.*, 2023 ▸; Soghomonyan *et al.*, 2016 ▸). To date, biomedical research using THz technologies has mainly focused on two aspects, using the characteristics of THz spectral properties to obtain physical parameters, and irradiation of biological samples to study changes in biological properties and post-exposure effects for safety (Liu *et al.*, 2023 ▸).

**Figure 1:**
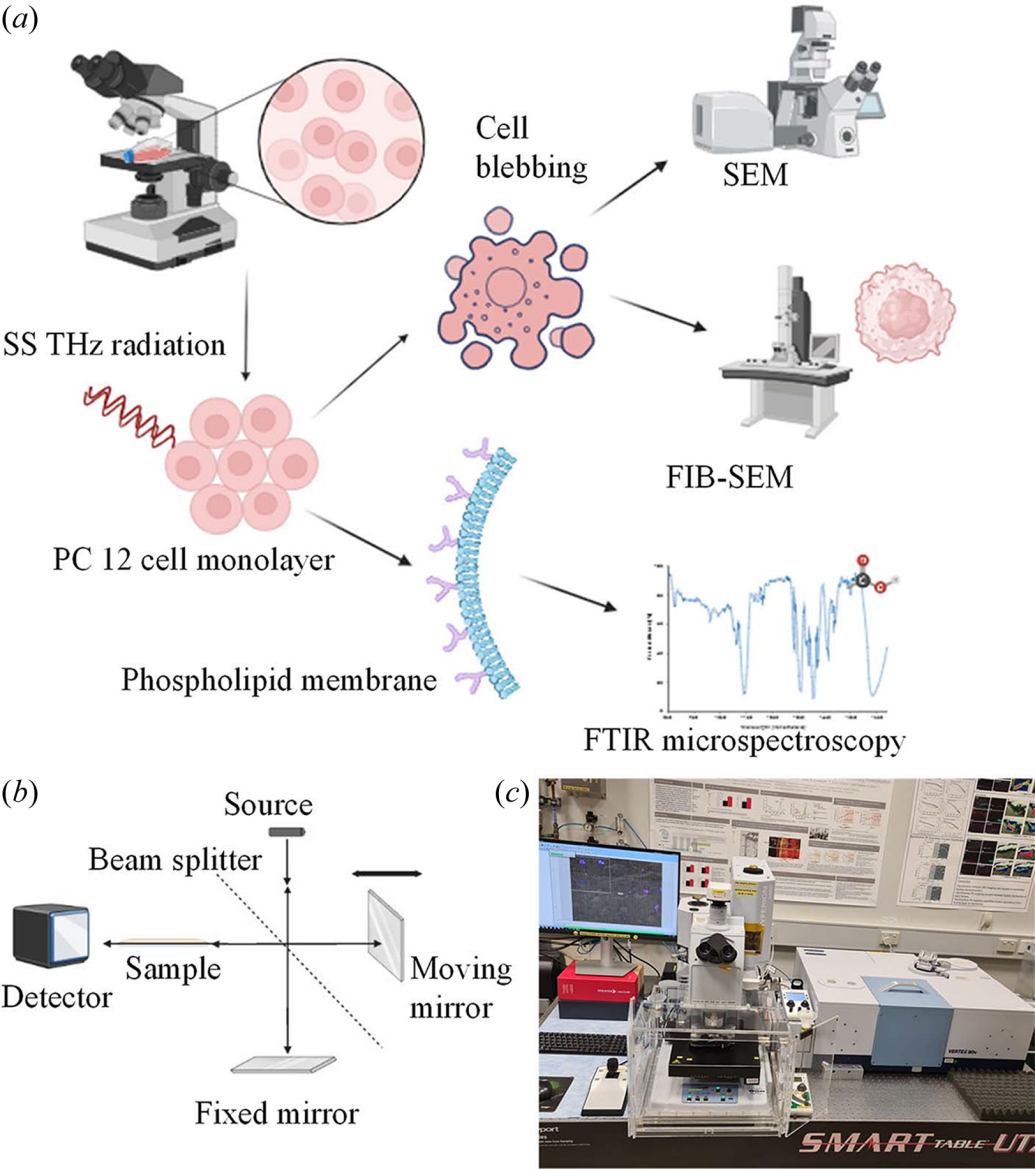
Experimental design and the technical setup at the Australian Synchrotron IRM beamline. (*a*) Following 10 min of SS THz exposures, the PC 12 cells were analysed for morphological changes at the membrane level including cell blebbing and membrane permeability. (*b*) Principle behind FTIR microspectroscopy: a beam consisting of IR wavelengths is sent through a beam splitter, where it is reflected via the mirrors and recombined to construct an interference pattern which is then sent to the sample and the transmitted portion reaches the detector. Fourier transformation is then performed to obtain a full spectrum as a function of wavenumbers. (*c*) FTIR microscope and spectrometer used on the IRM beamline at the Australian Synchrotron

Recently it was reported that high-frequency electromagnetic energy (EME) induced membrane permeabilization in different biological entities (Perera *et al.*, 2024 ▸) including PC 12 cells (Perera *et al.*, 2019*a* ▸,*b* ▸, 2018 ▸, 2022 ▸). It was shown that, following 10 min of SS THz irradiation, the PC 12 cells were able to internalize silica nanospheres that were found to be localized in the cytoplasm (22%), bound to the cell membrane (52%) or sequestered in vacuoles (26%) (Perera *et al.*, 2019*a* ▸, 2023 ▸). It was also found that the PC 12 cell morphology was altered through the formation of unusual large (0.5–2.0 µm in diameter) outer membrane blebbing (Fig. 1 ▸). The morphological changes after exposure to SS THz, however, did not compromise the viability of the PC 12 cells (Perera *et al.*, 2023 ▸). This observation prompted the current study, with the aim of identifying the key biochemical changes that follow SS THz radiation exposure.

## 2. Materials and methods

### 2.1. PC 12 cell culture and growth conditions

The PC 12 cell line used in this study was purchased from the American Type Culture Collection (ATCC, USA) and cultured in a complete Gibco RPMI medium (Thermo Fisher Scientific, Australia) supplemented with 10% Gibco horse serum (Thermo Fisher Scientific, Australia, HS), 5% Gibco foetal bovine serum (Thermo Fisher Scientific, Australia, FBS) and 1% Gibco penicillin/streptomycin (Thermo Fisher Scientific, Australia, PS). Supplements were stored as aliquots at −20°C. Stock solutions of the PC 12 cells were prepared in a medium containing 90% FBS and 10% DMSO and stored in liquid nitro­gen. The cells were maintained at 37°C with 5% CO_2_ in a 95% humidified incubator. The medium was changed every two days and passaged accordingly when the confluence reached 90%.

### 2.2. SS THz exposure at the beam extraction port

The Terahertz/Far-infrared (THz/Far-IR) beamline at the Australian Synchrotron (Victoria, Australia) has been adapted to serve as the THz exposure source, where samples can be placed in the path of the radiation at the beam extraction port (BEP). With this technique, the configuration of the SS THz beam utilized for the exposure delivers energies in the range 0.058 mW over a 9 mm radius spot size, giving an incident wideband power density of ∼1.25 W m^−2^ (0.125 mW cm^−2^).

The synchrotron has a 500 MHz frequency THz beam, which results in a THz ‘bundle’ spaced every 60 cm. Each ‘bundle’ takes up ∼8.8 mm, lasting ∼28.6 ps. It is a Gaussian function, in form. The average measured power at the extraction port was 1.25 W m^−2^; after some further losses, it was 1.0 W m^−2^ incident on the sample. The estimated peak incident power density at the extraction port is ∼200 W m^−2^ (about the energy intensity of light on a cloudy day).

At this power intensity, the maximum instantaneous heating at the surface, given a 200 W m^−2^ source for 5 ps and a water based sample (4.186 kJ kg^−1^ heat capacity), is given by
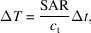
where Δ*T*_t_, *c*_t_ and Δ*t* are the temperature rise, heat capacity and length of time under consideration, respectively, and SAR is the specific absorption rate, given by
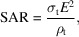
where *E*, σ_t_ and ρ_t_ are the maximum electric field (*E* field) strength, electrical conductivity and sample density, respectively. With *E*^2^ = power density × 377, 200 Sm^−1^ electrical conductivity, 1000 kg m^3^ density and 4.186 kJ kg^−1^ heat capacity, the maximum temperature rise after 5 ps at 200 W m^−2^ is on the order of 1.8 × 10^−10^°C.

The frequency of the beam is centred on 4.0 T Hz, with a half-maximum at approximately 2.0 T Hz and 8.0 T Hz, with a long, low-intensity tail into the infrared spectral region beyond 20 T Hz (Vilagosh *et al.*, 2022 ▸; Perera *et al.*, 2023 ▸). There is a rapid falloff in intensity below 1.5 T Hz. The exposure could be maintained for any arbitrary length of time, the time being limited only by the sample integrity and viability.

The PC 12 cells were exposed for 10 min to SS THz radiation at the BEP location. The change in temperature was negligible given that it was within ±0.4°C. The PC 12 cells were exposed for a period of 10 min as the time of exposure was limited by the sample integrity and viability. Our previous studies on the permeability of PC 12 cell membranes confirmed that 10 min of exposure had no detrimental effects to cell viability and hence we used the same exposure time in this work to identify possible changes that might be associated with the cell permeability following SS THz exposure. A monolayer of PC 12 cells was constructed at a concentration of 7.7 × 10^6^ cells ml^−1^ in 30 µl phosphate buffered saline on a polyethyl­ene film using an O-ring and sealed using vacuum grease. There are loses due to the sample holder, reducing the intensity to 1.0 W m^−2^. Water and water-containing samples such as aqueous solutions and cells have an absorption coefficient of 235 cm^−1^ at 1.0 T Hz and 3350 cm^−1^ at 20.0 T Hz. This results in most of the radiation being absorbed in the first 3 µm to 10 µm of the sample. Given that the PC 12 cells formed a monolayer at the interface between the sample holder and the beam, it was assumed that the cells were exposed to the full power of the incident beam. The total energy delivered to the sample over 10 min was estimated to be 600 J m^−2^.

### 2.3. SS FTIR microspectroscopy

This study was conducted at the Infrared Microspectroscopy (IRM) beamline of the Australian Synchrotron branch of ANSTO (Clayton, Victoria). SS FTIR microspectroscopic measurements were performed on the PC 12 cells in transmission mode at the IRM beamline using a Bruker Vertex 80V spectrometer coupled with a Hyperion 3000 FTIR microscope and a liquid-nitro­gen-cooled narrow-band mercury cadmium telluride detector. A matching pair of 36× objective and condenser (NA = 0.50; Bruker Optik GmbH, Ettlingen, Germany) was used to acquire chemical maps of the THz-exposed PC 12 samples that were fixed using a cocktail of 4.0% paraformaldehyde and 2.5% glutaraldehyde for 30 min. After fixation, the cell solution was washed twice before placing 10 µl on a CaF_2_ window.

In this study, single-point spectra were collected on individual cells with a beam-defining aperture that provided a nominal measurement area of 6.9 µm in diameter and 128 co-added scans. All the SS FTIR spectra were recorded within the wavenumber range 3800–950 cm^−1^ using a spectral resolution of 4 cm^−1^. Default acquisition parameters, including Blackman–Harris 3-Term apodization, power-spectrum phase correction and a zero-filling factor of two were set using the *OPUS 8* software suite (Bruker). Background spectra were collected using the same acquisition parameters on a clean area of the same CaF_2_ window with no PC 12 cells.

### 2.4. Spectral pre-processing and principal component analysis

SS FTIR spectra of the single cells obtained from both control and THz-exposed groups were individually examined, and those that presented weak absorption intensities with a low signal-to-noise ratio were manually removed from the datasets. The spectra that passed this initial screening test were then combined into one dataset and pre-processed by calculating second derivatives using nine-point smoothing with the Savitzky–Golay algorithm to eliminate broad baseline offset and curvature as well as to enhance the features of hidden and overlapping bands. Prior to the principal component analysis (PCA), extended multiplicative signal correction (EMSC) was applied to the resultant second-derivative spectra within the two spectral windows (*i.e.* wavenumbers 3055–2800 cm^−1^ and 1810–1000 cm^−1^). These spectral ranges contain the key biochemical information relevant to most biological samples involving proteins, lipids, carbohydrates and nucleic acids. In principle, the EMSC pre-treatment is commonly used to remove any light-scattering artefacts and to normalize the spectra, accounting for differences in path length. Therefore, using EMSC-corrected second-derivative spectra essentially yields the results that correspond in a more linear fashion to the analyte concentration compared with the results obtained using untreated spectra. This pre-processing approach has reportedly led to greater interpretability and an improved discriminatory accuracy of the multivariate data analysis approaches including the PCA performed in this study (Vongsvivut *et al.*, 2015 ▸, 2013 ▸).

PCA was subsequently applied on the combined EMSC-corrected second-derivative spectral dataset within the same spectral regions used previously for the EMSC (*i.e.* wavenumbers 3055–2800 cm^−1^ and 1810–1000 cm^−1^) and full cross-validation method, using The *Unscrambler X* software package (version 10.3; CAMO Software AS, Oslo, Norway). In this study, the PCA approach was performed to determine specific functional groups relevant to the key cellular constituents that influence the differentiation between the SS THz-exposed PC 12 cells and the unexposed control cells, to gain a better understanding of the impact of THz exposure at the single-cell level.

### 2.5. Statistical analysis

Statistical data processing was conducted using the *Statistical Package for the Social Sciences* (*SPSS*, version 24.0 Chicago, IL, USA). Statistically significant differences (*p* < 0.05) among the various groups were calculated using the independent groups *t*-test, where the independent variable was the treatment condition.

## 3. Results and discussion

### 3.1. FTIR spectral results and PCA analysis of SS THz-exposed PC 12 cells

To realize which of the major classes of biomolecules might be susceptible to high-frequency EME in the SS THz range in particular, analysis of the SS FTIR spectra was conducted for individual cells. An overview of the experimental design and technical setup at the IRM beamline is illustrated in Fig. 1 ▸.

Our subsequent analysis is based on the detailed band assignments given in Table 1 ▸. In general, spectral features in the wavenumber range 3100–2800 cm^−1^ correspond to the ν(C—H) stretching vibrations of methyl­ene and methyl groups of lipids. Specifically, the two pairs of peaks at 2960/2872 cm^−1^ and 2925/2852 cm^−1^ can be attributed to the asymmetric/symmetric stretches of ν(C—H) vibrations from methyl (–CH_3_) and methyl­ene (–CH_2_) functional groups, respectively (Guillén & Cabo, 1997 ▸). It was reported that that major lipids found in PC 12 cells were cholesterol (13%), phosphatidyl­ethano­lamine (22–24%) and phosphatidylcholine (34–39%) (Traynor *et al.*, 1982 ▸; Ariga *et al.*, 1988 ▸). Extended analysis of lipid composition of PC 12 cell membranes indicated that globoside is the predominant neutral glycolipid present in PC 12 cells. Globoside has also been reported to be present in the plasma membrane of sympathetic neurons cultured from new-born rats; an important characteristic of globoside is that it contains saturated normal fatty acids of variable chain length (Ariga *et al.*, 1988 ▸).

**Figure 2:**
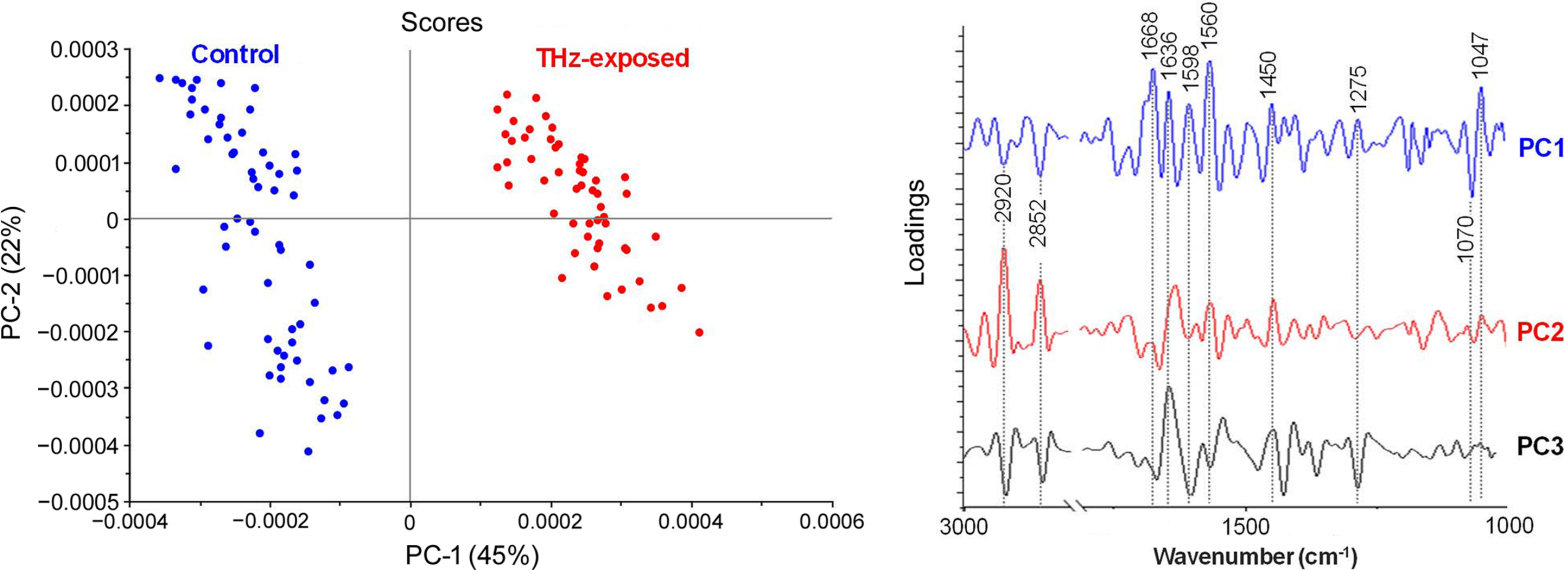
Comparative PCA analysis of PC 12 cells exposed to SS THz radiation. The scores plot (left) reveals the cluster separation between the exposed and control cells against the first two PCs, and the loadings plot (right) shows the absorption peaks with the most influence on the separation. The detailed band assignment based on the position of peak minima found in the second-derivative spectra and the corresponding references are given in Table 1 ▸.

Based on the PCA analysis of the SS FTIR spectra in Fig. 2 ▸ and the detailed FTIR band assignments in Table 1 ▸, the scores plot clearly shows a distinct clustering pattern of these single cells that separated the SS THz-exposed group from the non-exposed control group along PC1. According to the feature of the PC1 loadings, the main contribution to the variation in these two cell groups arises from the protein compositions, which are observed as positive loadings, suggesting higher proportions of various protein structures in the control group; previous findings also confirmed a higher total protein content in the control samples in comparison with the exposed samples (Perera *et al.*, 2019*a* ▸). These include the amide I bands at wavenumbers 1668 cm^−1^ and 1636 cm^−1^ (*i.e.* β-turn and antiparallel β-sheet, respectively), the tyrosine band at 1598 cm^−1^, and the amide II bands at 1560 cm^−1^ (*i.e.* perpendicular modes of the α-helix and antiparallel β-sheet), as well as δ(CH_3_) of proteins at 1450 cm^−1^. The SS THz-exposed cells, on the other hand, showed a slight yet noticeable increase in lipids, specifically cholesterol, as shown through the negative loadings at wavenumbers 2920 cm^−1^ and 2852 cm^−1^ [*i.e.* asymmetric and symmetric ν(C—H) stretching modes of methyl­ene (–CH_2_) groups in the lipid structures], which align with the CH_2_ and CH_3_ peaks characteristic of the asymmetric stretching present in cholesterol. In addition, the nucleic acid components were found to have a significant impact on the THz-exposed cells, as shown through the presence of the negative loadings at 1070 cm^−1^, attributable to the νs(PO_2_–) stretching vibration of the phosphate groups that can be found in the phospho­diester backbone of nucleic acids (DNA and RNA) and phospho­lipid membranes.

It is suggested that cholesterol has a peak around 2925 cm^−1^, corresponding to CH_2_ and CH_3_ asymmetric stretching (Gupta *et al.*, 2014 ▸), which is a ubiquitous component of mammalian cell membrane (Sliskovic & White, 1991 ▸), and is known to play a crucial role in influencing the physical and chemical properties of the cell membrane, which includes the regulation of membrane fluidity, permeability and adjustments of the lateral mobility (Gupta *et al.*, 2014 ▸). Cholesterol, along with sphingolipids, is known to be involved in transient membrane interactions via the formation of raft-like structures to regulate immune signalling, cancer and cardiovascular diseases (Bagheri *et al.*, 2020 ▸). Therefore, we propose that cholesterol molecules might play a role in modulation of membrane fluidity in PC 12 cells during exposure to EME THz radiation.

Exposure to SS THz radiation shown previously has been re-confirmed in this study using the cells from the same samples used for recording the SS FTIR spectra, to induce cell membrane permeabilization in PC 12 (Fig. S1 of the supporting information) (Perera *et al.*, 2019*b* ▸, 2023 ▸). In addition to cell membrane permeabilization, nanosphere localization following SS THz radiation was further confirmed using complementary electron microscopy techniques (Fig. S1). Apart from membrane permeability, the cells exhibited the formation of large blebs on the outer membrane in response to SS THz radiation, as shown using scanning electron microscopy, transmission electron microscopy and focused-ion beam scanning electron microscopy (Fig. S2) (Perera *et al.*, 2023 ▸). It was shown that the PC 12 cells remain viable and physiologically healthy based on the results of bioassays assessing membrane integrity, protein concentration and metabolic activity (Perera *et al.*, 2019*a* ▸; Perera, 2020 ▸). In our previous work, we reported that the post-exposure long-term analysis of the PC 12 cells showed that the PC 12 cells did not exhibit significant differences in their metabolic activity in comparison with non-exposed controls (Perera *et al.*, 2019*a* ▸; Perera, 2020 ▸). In fact, a higher proportion of the population of PC 12 cells exhibited neuronal differentiation in comparison with the unexposed control sample (Perera *et al.*, 2019*a* ▸; Perera, 2020 ▸).

While PC 12 and other eukaryotic cells have been found to form small blebs 0.5–1 µm in diameter, to date the large blebs 1–2 µm in diameter have only been reported for PC 12 after exposure to SS THz (Perera *et al.*, 2023 ▸). It was established that the blebbing formation is a dynamic process facilitated by remodelling the sub-membranous cytoskeleton which, in turn, leads to reorganization of the plasma membrane (Fackler & Grosse, 2008 ▸). Rearrangements of the cytoskeleton and downstream signalling cascades result in distinct types of actin-rich invaginations or protrusions including podosomes, lamellipodia, filopodia and blebs (Fackler & Grosse, 2008 ▸; Chhabra & Higgs, 2007 ▸). Blebs can be defined as spherical expansions of the outer membrane, protrusions away from the cell membrane devoid of filamentous actin (Paluch & Raz, 2013 ▸) which can be identified on a two-dimensional scale [Figs. S2(*a*) and S2(*d*)]. Interestingly, the lifetime of a bleb is approximately 1 min: it initiates with expansion followed by a short static phase to a low retraction of the bleb to the exact outer membrane location where it originated from (Charras *et al.*, 2005 ▸; Tournaviti *et al.*, 2007 ▸). It was suggested that blebbing arises as a result of internal hydro­static pressure (Fackler & Grosse, 2008 ▸) and is highly dependent on filamentous actin integrity (Fackler & Grosse, 2008 ▸).

Indeed, analysis of the bands of the FTIR spectra that appear in the wavenumber ranges 1680–1630 cm^−1^, 1560–1510 cm^−1^ and 1260–1220 cm^−1^ indicate that these bands arise due to the presence of proteins and specifically due to amide I, II and III modes, respectively (Böcker *et al.*, 2007 ▸). Among the peaks for the amide bands in proteins, the spectral bands most sensitive to variations in the secondary structure of proteins appear to be amide I and II. It is likely that detected variations in the secondary structure might correspond to changes in the secondary structure of actin-related proteins. This class of proteins is responsible for the maintenance of the cells’ cytoskeleton, which is a highly dynamic network composed of actin and related proteins (Schmidt & Hall, 1998 ▸; Svitkina, 2018 ▸). The actin cytoskeleton is also responsible for mediating cell motility and shape changes during the cell cycle and in response to external stimuli assisting the cytoplasm in generating mechanical forces within the cell (Schmidt & Hall, 1998 ▸; Svitkina, 2018 ▸). FTIR spectra of actin reported in previous studies have shown the predominant band at 1653 cm^−1^ attributed to the α-helix conformation (Gicquaud & Wong, 1994 ▸) along with another band at 1651 cm^−1^ attributed to the β-sheets, confirming the secondary structure of proteins; the α-helices appear to be the predominant secondary structure of actin (Gicquaud & Wong, 1994 ▸). Here, the distinct peak at 1660 cm^−1^ suggests the dominance of the β-turn with lower peaks around 1654 cm^−1^ confirming the presence of α-helical proteins, in agreement with previous reports (Gicquaud & Wong, 1994 ▸). Thus, the formation of blebs in PC 12 cells in response to SS THz radiation could be due to the effect of high-frequency EME on the secondary structure of actin-related molecules, as shown previously for peptides (Todorova *et al.*, 2016 ▸) to be the result of SS THz radiation with regards to membrane blebbing (St-Onge & Gicquaud, 1989 ▸).

In PC 12 cells exposed to SS THz, we observed changes with respect to negative loadings present at 1070 cm^−1^ that might be attributable to the ν_s_(PO_2_–) stretching vibration of the phosphate groups that can be found in the phospho­diester backbone of nucleic acids (DNA and RNA) (Whelan *et al.*, 2011 ▸). Further experimental analysis is required to conclude on the direct effects on DNA which will be investigated in detail.

## 4. Conclusions

Synchrotron-FTIR analysis of PC 12 cells exposed to SS THz radiation revealed that the possible changes in biomolecules such as cholesterol, which is abundantly present in membrane lipids and actin-related proteins in the cytoskeleton, might contribute to reversible membrane permeabilization and outer membrane blebbing. However, further work is required to identify the nature of the changes in molecular organization of biomolecules.

## Supplementary Material

Materials and methods, additional data on PC 12 cells permeabilisation and cell blebbing, and 2D PCA scores. DOI: 10.1107/S1600577524010944/ing5002sup1.pdf

## Figures and Tables

**Table 1:** FTIR band assignment for functional groups found in the second-derivative spectra of PC 12 cells

Wavenumber (cm^−1^)	Band assignment†	Reference
∼3014	ν(C—H) of *cis* C=CH–	Guillén & Cabo (1997 ▸)
∼2960	ν_as_ (C—H) from methyl (–CH_3_) groups of lipids	Tammer (2004 ▸)
∼2925	ν_as_ (C—H) from methyl­ene (–CH_2_) groups of lipids	Tammer (2004 ▸)
∼2852	ν_s_ (C—H) from methyl­ene (–CH_2_) groups of lipids	Tammer (2004 ▸)
∼1670	Amide I: β-turn	Böcker *et al.* (2007 ▸)
∼1654	Amide I: α-helix	Böcker *et al.* (2007 ▸)
∼1638	Amide I: antiparallel β -sheet	Guillén & Cabo (1997 ▸)
∼1598	Tyr or *p*-cresol	Barth (2007 ▸)
∼1560	Amide II: perpendicular modes of the α-helix and antiparallel β sheet	Miyazawa & Blout (1961 ▸)
∼1514	Amide II: parallel mode of the α-helix	Miyazawa & Blout (1961 ▸)
∼1450	δ_as_(CH_3_) of proteins (possibly in DNA and RNA)	
∼1382	δ_s_(CH_3_) and δ_s_(CH_2_) of lipids and proteins	Guillén & Cabo (1997 ▸)
∼1369	δ_s_(CH_3_) from methyl groups of cholesterol and fatty acid radicals	Chapman (1965 ▸)
∼1310	Amide III: α-helix	Cai & Singh (1999 ▸)
∼1264	ν_s_ (C—O) and/or δ(O—H) possibly of carb­oxy­lic acids	Sills *et al.* (1994 ▸), Smidt *et al.* (2005 ▸)
∼1172	ν_s_ (C—O—C) from esters	Böcker *et al.* (2007 ▸)
∼1155	ν_as_ (CO—O—C) of glycogen and nucleic acids (DNA and RNA)	Guillén & Cabo (1997 ▸)
∼1045	ν(C—O) coupled with δ(C—O) of C—OH groups of carbohydrates	Wong *et al.* (1991 ▸)
∼1025	ν(C—C)_skeletal_ coupled with δ(CH_2_) of α-CH_2_ in –CH_2_OH groups of polysaccharides	Chapman (1965 ▸), Wong *et al.* (1991 ▸)

† ν_as_ = asymmetric stretch, ν_s_ = symmetric stretch, δ_s_ = symmetric in-plane deformation (bend), δ_as_ = asymmetric in-plane deformation (bend).
