# Supplementary material for: Shedding light on biochemical changes in single neuron-like pheochromocytoma cells following exposure to synchrotron sourced terahertz radiation using synchrotron source Fourier transform infrared microspectroscopy
Source: J Synchrotron Radiat. 2025 Jan 1;32(Pt 1):155–61. doi: 10.1107/S1600577524010944 (PMC11708867; doi:10.1107/S1600577524010944)
Supplement: Supplementary file 1 [file s-32-00155-sup1.pdf]

JOURNAL OF SYNCHROTRON RADIATION

Volume 32 (2025)

Supporting information for article:

Shedding light on biochemical changes in single neuron-like pheochromocytoma cells following exposure to synchrotron sourced terahertz radiation using synchrotron source Fourier transform infrared microspectroscopy

Palalle G. Tharushi Perera, Jitraporn Vongsvivut, Denver Linklater, Zoltan Vilagosh, Dominique Appaddo, The Hong Phong Nguyen, Mark Tobin, Rodney Croft and Elena P. Ivanova

1. Materials and Methods

1.1. Internalisation of silica shelled gold nanospheres

Silica core-shell gold nanospheres with a diameter of 50 nm ± 5 nm (nanoComposix, San Diego, CA) were used to study the uptake and localisation of PC 12 cells. Immediately following SS THz exposure, the nanospheres were added into the cell suspension at a concentration of 10 µg/mL. After 5 min of incubation, the samples were washed twice using PBS and centrifuged at 1300 rpm for 5 min at 25 °C. The procedure was repeated for the untreated controls, where the cell samples were mixed with 10 µL of AuSi NS.

1.2. Transmission Electron Microscopy

After 5 min of incubation in the presence of nanospheres following SS THz exposure, cell suspensions were pelleted by centrifugation at 1300 rpm for 5 min at 25 °C. The cells were then washed twice with phosphate buffer saline (PBS, 10 mM, pH 7.4) to remove any unbound nanospheres. The cell pellet was conditioned with 0.1M sodium cacodylate buffer (pH 7.4). The cell pellet was then re-suspended in primary fixative of 4 % paraformaldehyde and 2.5% glutaraldehyde in 0.1M sodium cacodylate buffer overnight at 4 degrees and washed thrice in cacodylate buffer for 10 minutes each. The cells were post-fixed in 1% osmium tetroxide (OSO4) and 1.5 % potassium ferrocyanide for 1 h followed by three washes in distilled water for 10 mins each. The cells were dehydrated using a graded series of ice-cold ethanol (50%, 70%, and 90%) for 15 min each. The cells were further dehydrated by passing through 100% ethanol twice followed by 100% acetone twice for 30 min each. The cells were further infiltrated with 1:1 ratio of acetone: Spurr’s resin mixture for overnight. After that the cells were completely exchanged in 100% Spurr’s resin twice for 3 hrs each time, under vacuum. The resin samples were polymerised at 70 °C for 48 h. The final block was trimmed, then cut into ultrathin sections (90 nm thickness) using a Leica Ultracut Ultramicrotome (Leica Microsystems, Wetzlar, Germany) with a diamond knife (Diatome, Pennsylvania, USA). Sections were placed onto 200 mesh copper grids and examined using a JEM 1010 instrument (JEOL). Approximately 40 TEM images were taken at ×5000 and ×10000 magnifications for sample analysis.

1.3. Focused Ion Beam- Scanning Electron Microscopy

SS THz exposed PC12 cells were embedded as described in Section 2.4. A 400 x 400 µm block was trimmed by ultramicrotomy and affixed to an aluminium stub with silver paint. The block was sputter coated with 10 nm of iridium prior to loading in an FEI SCIOS dual beam focused ion beam scanning electron microscopy (FIB-SEM) system. A cross-section was milled in the block using a gallium ion beam at 0.1 nA and 30kV. Serial sections were milled and imaged using the Auto Slice and View 4

software (ThermoFisher). The images were aligned with Image J and 3D volumes were reconstructed using Slicer 3D (version 5.2.2).

1.4. Cellular morphology

The scanning electron microscope FeSEM SUPRA 40VP (Carl Zeiss, Jena, Germany) with a primary beam energy of 3 kV was used. A 100 µL aliquot of cells in PBS were placed on a glass cover slip (ProSciTech, Kirwan, Australia) in duplicate. The glass cover slips were then washed with nanopure H2O (resistivity of 18.2 MW cm-1) and dried with 99.99% purity nitrogen gas. The PC 12 cells exposed to 10 min of SS THz radiation were fixed in a cocktail of 2.0 % paraformaldehyde and 2.5 % glutaraldehyde for 30 min. The cells were then dehydrated by passing through a graded ethanol series (20 %, 40 %, 60 %, 80 % and 100 %) for 15 min. Before imaging, the fixed cells were subjected to gold sputtering (7 nm thick) using a NeoCoater MP-19020NCTR (JEOL, Tokyo, Japan). The same procedure was applied to non - treated PC 12 cells.

1.5. Cell viability

The viability of PC 12 cells was determined using the LIVE/DEAD Viability/Cytotoxicity Kit (Invitrogen) according to the manufacturer protocol. The viability of the SS THz irradiated cells and the controls was monitored immediately after the treatment and confirmed through three technical replicates. CLSM was used in assessing the number of viable cells; approximately 10 fields of view were analysed per sample type and the number of cells per mm2 were expressed.

SS THz induced transient membrane permeabilisation in PC 12 cells

Membrane permeabilisation was confirmed in PC 12 cells using TEM where Au Si NPs (Figure S1) and FITC NS [1] were internalised following a 10 min exposure of SS THz radiation, Au Si NS localisation was further confirmed using scanning transmission electron microscopy energy-dispersive spectroscopy (STEM-EDS) [2]. Viability of PC 12 cells following exposure to SS THz radiation appears to be similar to that of the unexposed control sample (Figure S1e-f) and it has been previously quantified and confirmed that the viability of the cells remain unaffected [1, 3]. The morphology analysis following SS THz exposures have revealed that the OM of PC 12 cells exhibited membrane blebbing or protrusions of the membrane (yellow arrow) as confirmed previously [3].

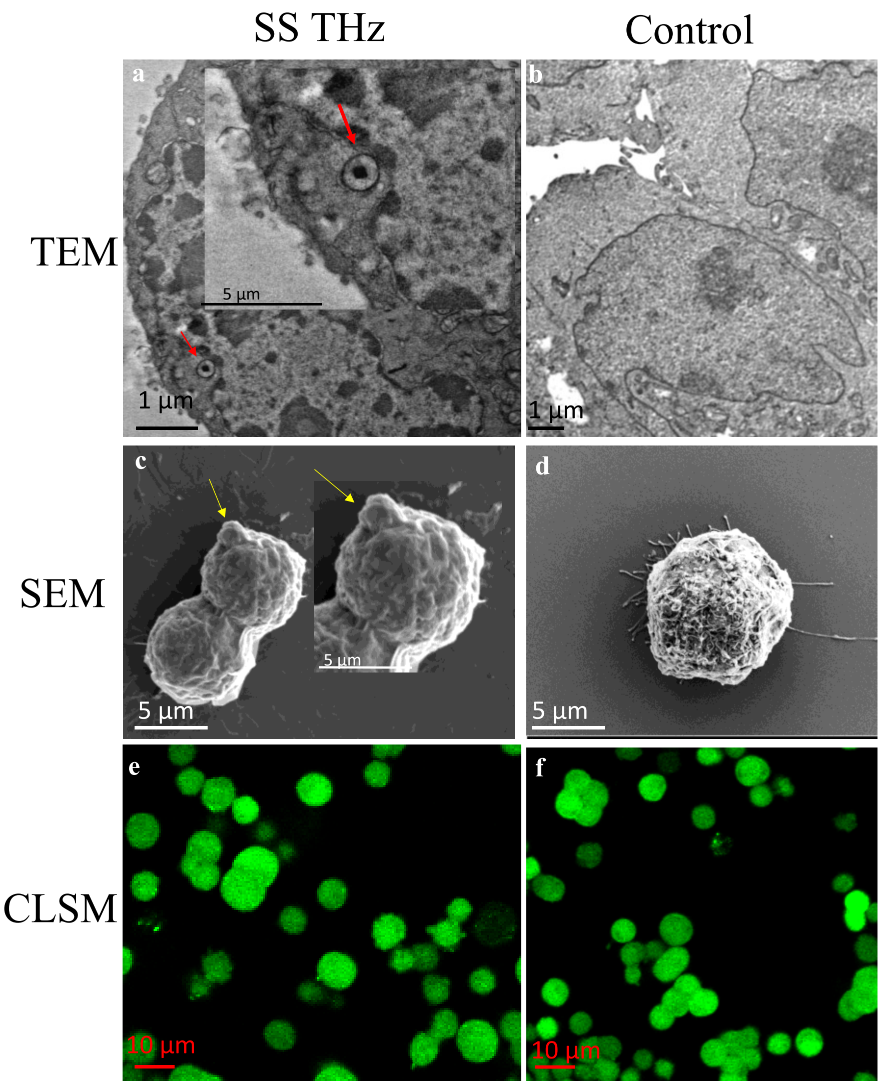

Figure S1 Synchrotron Sourced THz -induced NS uptake by PC 12 cells and morphological changes. (a) TEM micrographs displaying AuSi NS uptake (red arrows), NS appear to be localised inside the cytoplasm (insets) and closer to the cell membrane (b) unexposed PC 12 control sample, (c) SEM micrographs of PC 12 exhibiting cell membrane blebbing (insets) (d) absence of cell blebbing in the unexposed sample (e) PC 12 cells remain viable post exposure to SS THz radiation of 10 min, similar to that of the control sample (f).

SS THz induced blebbing in PC 12 cells

Further analysis of PC 12 cell membrane was carried out using various electron microscopy techniques (Figure 3a-d), to confirm cell outer membrane blebbing using SEM imaging (Figure S2a) and STEM-EDS imaging (Figure S2b) (circled in green) as further confirmed using FIB SEM (Figure S2c-d). The volume construction of the blebbing is illustrated in green.

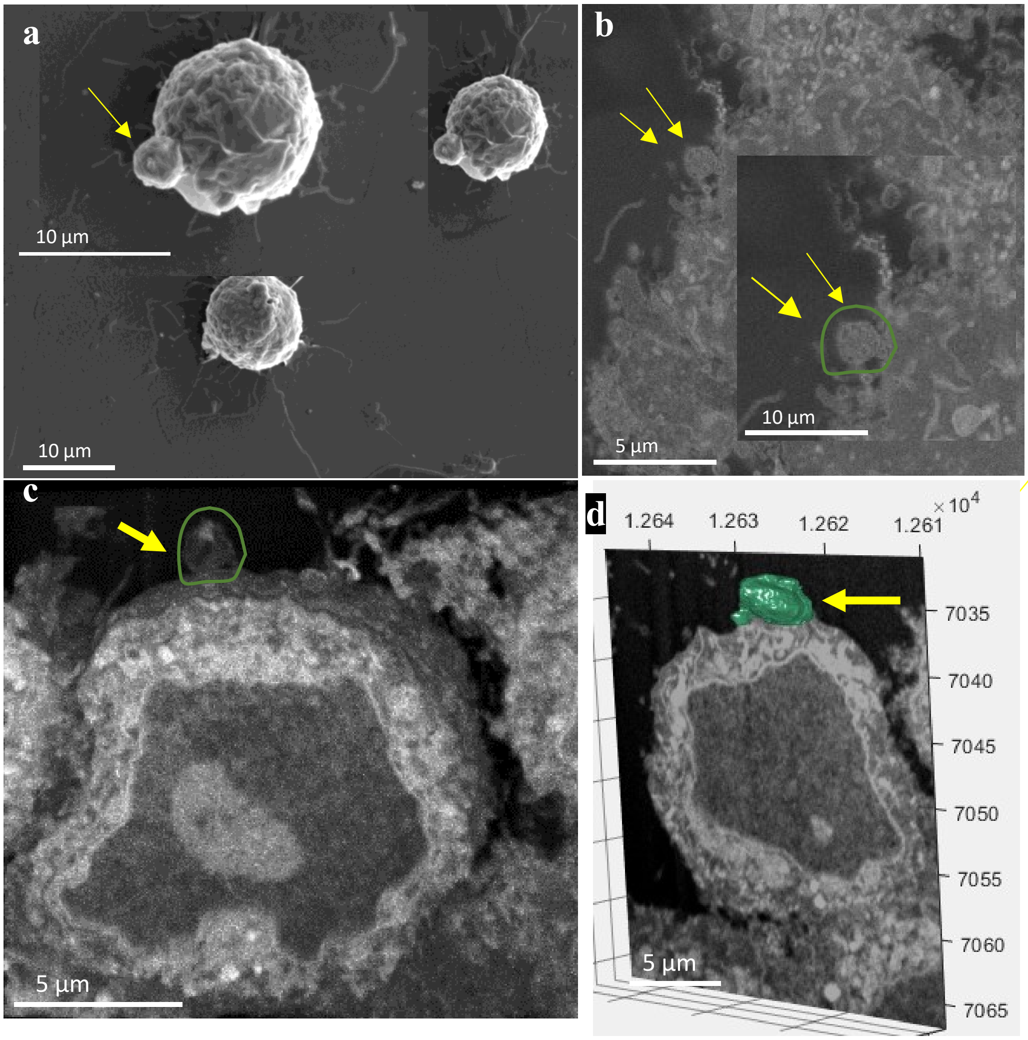

Figure S2. Analysis of bleb formation in PC 12 in response to SS THz using electron microscopy. (a) Bleb formation visualised using SEM (yellow arrows; insets), (b) STEM-EDS image of a PC 12 where the blebs are evident in thinly sliced synchrotron exposed PC 12 cells (c) FIB-SEM of PC 12 cells, the blebs appear as protrusions of the cell membrane (circled in green) (d) volumetric 3D makeup of blebs (highlighted in green), different EM techniques confirming their presence post exposure.

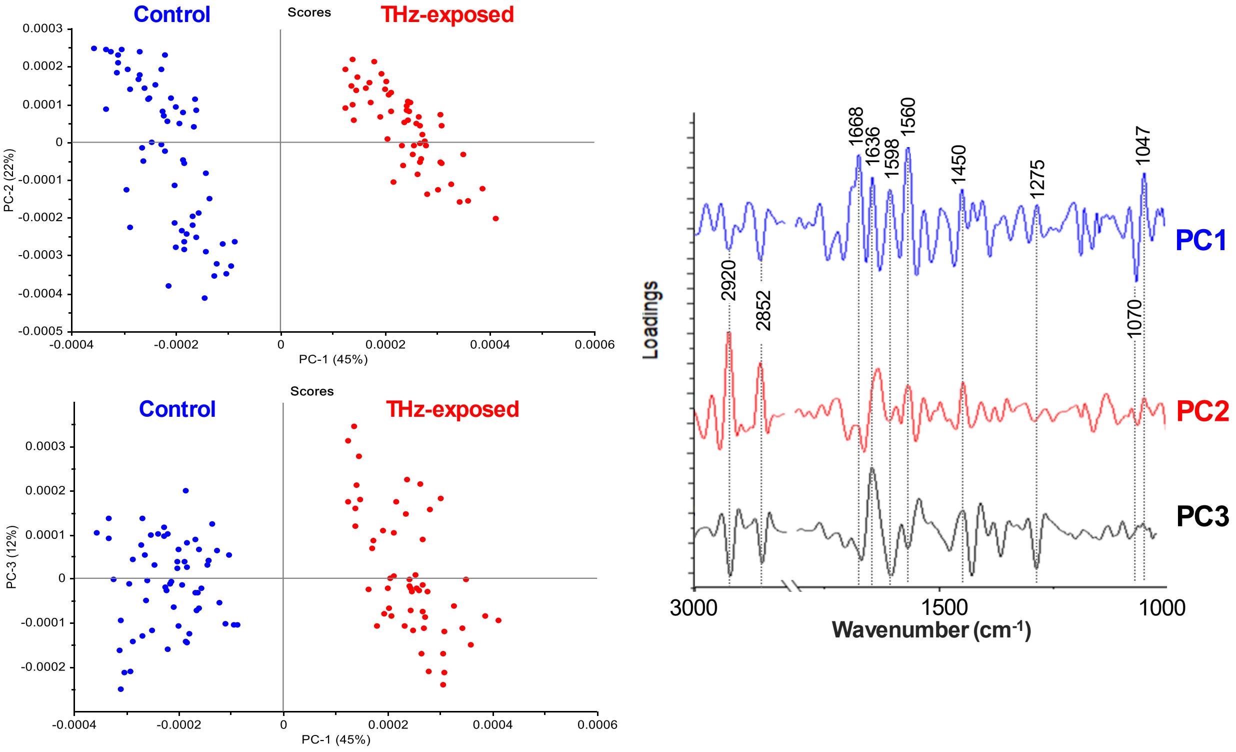

Figure S3. 2D PCA scores plots showing the separation of the THz exposed cells and control cells along the PC-1 axis along with the loadings.

References

1. Perera, P.G.T., et al., *PC 12 Pheochromocytoma Cell Response to Super High Frequency Terahertz Radiation from Synchrotron Source.* Cancers, 2019. **11**(162): p. 1-17.
2. Perera, P.G.T., et al., *Translocation and fate of nanospheres in pheochromocytoma cells following exposure to synchrotron-sourced terahertz radiation.* Journal of Synchrotron Radiation, 2023. **30**(Pt 4): p. 780-787.
3. Perera, P.G.T., et al., *Exposure to high-frequency electromagnetic field triggers rapid uptake of large nanosphere clusters by pheochromocytoma cells.* International Journal of Nanomedicine, 2018. **13**: p. 8429-8442.
